# Insights into the Genetic Relationships and Breeding Patterns of the African Tea Germplasm Based on nSSR Markers and cpDNA Sequences

**DOI:** 10.3389/fpls.2016.01244

**Published:** 2016-08-30

**Authors:** Moses C. Wambulwa, Muditha K. Meegahakumbura, Samson Kamunya, Alice Muchugi, Michael Möller, Jie Liu, Jian-Chu Xu, Sailesh Ranjitkar, De-Zhu Li, Lian-Ming Gao

**Affiliations:** ^1^Key Laboratory for Plant Diversity and Biogeography of East Asia, Kunming Institute of Botany, Chinese Academy of SciencesKunming, China; ^2^Germplasm Bank of Wild Species in Southwest China, Kunming Institute of Botany, Chinese Academy of SciencesKunming, China; ^3^College of Life Science, University of Chinese Academy of SciencesKunming, China; ^4^Genetic Resources Unit, World Agroforestry CentreNairobi, Kenya; ^5^Tea Research Institute, Kenya Agricultural and Livestock Research OrganizationKericho, Kenya; ^6^Genetic and Plant Breeding Division, Coconut Research InstituteLunuwila, Sri Lanka; ^7^Department of Science, Royal Botanic Garden EdinburghEdinburgh, UK; ^8^Centre for Mountain Ecosystems, Kunming Institute of Botany, Chinese Academy of SciencesKunming, China

**Keywords:** African tea germplasm, breeding improvement, *Camellia sinensis*, genetic diversity, nSSR markers, cpDNA regions

## Abstract

Africa is one of the key centers of global tea production. Understanding the genetic diversity and relationships of cultivars of African tea is important for future targeted breeding efforts for new crop cultivars, specialty tea processing, and to guide germplasm conservation efforts. Despite the economic importance of tea in Africa, no research work has been done so far on its genetic diversity at a continental scale. Twenty-three nSSRs and three plastid DNA regions were used to investigate the genetic diversity, relationships, and breeding patterns of tea accessions collected from eight countries of Africa. A total of 280 African tea accessions generated 297 alleles with a mean of 12.91 alleles per locus and a genetic diversity (*H*_S_) estimate of 0.652. A STRUCTURE analysis suggested two main genetic groups of African tea accessions which corresponded well with the two tea types *Camellia sinensis* var. *sinensis* and *C. sinensis* var. *assamica*, respectively, as well as an admixed “mosaic” group whose individuals were defined as hybrids of F2 and BC generation with a high proportion of *C. sinensis* var. *assamica* being maternal parents. Accessions known to be *C*. *sinensis* var. *assamica* further separated into two groups representing the two major tea breeding centers corresponding to southern Africa (Tea Research Foundation of Central Africa, TRFCA), and East Africa (Tea Research Foundation of Kenya, TRFK). Tea accessions were shared among countries. African tea has relatively lower genetic diversity. *C. sinensis* var. *assamica* is the main tea type under cultivation and contributes more in tea breeding improvements in Africa. International germplasm exchange and movement among countries within Africa was confirmed. The clustering into two main breeding centers, TRFCA, and TRFK, suggested that some traits of *C*. *sinensis* var. *assamica* and their associated genes possibly underwent selection during geographic differentiation or local breeding preferences. This study represents the first step toward effective utilization of differently inherited molecular markers for exploring the breeding status of African tea. The findings here will be important for planning the exploration, utilization, and conservation of tea germplasm for future breeding efforts in Africa.

## Introduction

Tea is one of the most popular beverages and has become a daily drink for billions of people across the world. The culture of drinking tea originated in China, and has been adopted in many parts of the world due to its numerous health benefits and attractive aroma (Yang et al., 2009; Bedran et al., 2015; Yin et al., 2015). The current global tea production stands at 5.35 million tons *Food and Agricultural Organization of the United Nations* (FAO, 2015a), with an estimated total sale of 10.84 billion U.S. dollars. Africa is one of the key centers for tea production, accounting for about 25% of the global black tea industry *Food and Agricultural Organization of the United Nations* (FAO, 2015a). The main tea producing countries in Africa include Kenya, Malawi, Uganda, Tanzania, Zimbabwe, South Africa, and Rwanda where tea contributes significantly to their respective economies. For instance, Kenya's tea exports earned the country around $1.33 billion in 2013 *Food and Agricultural Organization of the United Nations* (FAO, 2014). It is projected that the continent's export volume will reach 743,384 metric tons by the year 2023 (FAOSTAT, 2015).

The first commercial tea plantation in Africa was established in 1878 in Malawi near Blantyre (Anonymous, 1962). However, it was not until 1956 that breeding work would begin in Malawi following the establishment of the Tea Research Foundation of Central Africa (TRFCA; Ellis and Nyirenda, 1995). Clone establishment started with field selections (FS) from established seedling populations in Swazi, Malawi. The seedling populations had been established earlier using a few founder cultivars from China and India (Eastern Produce Malawi Ltd. Personal interview. 4 March 2015). Clones selected in this way were denoted SFS (Swazi Field Selection) e.g., SFS 204 and SFS 150. These were used as parental resources, which eventually gave rise to the present tea germplasm of southern Africa through crossing and further selection. A few years later in 1961, breeding work commenced in Kenya under the oversight of the Tea Research Institute of East Africa which was in 1980 renamed Tea Research Foundation of Kenya (TRFK; Mondal, 2014). Historical records show that seeds of tea plants from India and Sri Lanka were used to establish the pioneer tea plantations in Kenya in early 1900s (Matheson and Bovill, 1950). Because these progenies had not been particularly selected for high yield and quality, the resultant seedling populations of mixed genotypes were phenotypically inferior, though diverse. Tea cultivar improvement started by mass selecting among the introduced seedlings to develop pioneer cultivars such as TRFK 6/8.

The tea plant, *Camellia sinensis* (L.) Kuntze, is a woody evergreen plant in the family *Theaceae* and is native to the region covering the northern part of Myanmar, and the provinces of Yunnan and Sichuan in China (Wight, 1959). Based on morphological features, Wight (1962) defined three tea types of cultivated tea, i.e., *C*. *sinensis* var. *sinensis* (China tea), *C*. *sinensis* var. *assamica* (Masters) Hung T. Chang (Assam tea), and its subspecies rank *C*. *assamica* subsp. *lasiocalyx* (Planchon ex Watt.) Wight (Cambod tea). These groups were later treated as one species with two varieties [*C. sinensis* var. *sinensis* and *C. sinensis* var. *assamica* (J. W. Mast.) Kitam., including *C*. *assamica* subsp. *lasiocalyx* as a synonym; Ming, 2000; Ming and Bartholomew, 2007]. Our recent study indicated that *C*. *assamica* subsp. *lasiocalyx* is a hybrid between *C. sinensis* var. *sinensis* and *C. sinensis* var. *assamica*, and not a distinct genetic entity (Wambulwa et al., 2016), and the cultivated tea plant includes three distinct lineages (Meegahakumbura et al., 2016). Efforts have been made previously to assess the genetic diversity, relationships, and population structure of the tea germplasm in Africa (Wachira et al., 1995; Paul et al., 1997; Yao et al., 2008; Wambulwa et al., 2016). However, none of these studies attempted to characterize the entire African tea germplasm using robust molecular markers. For instance, Wachira et al. (1995) and Paul et al. (1997) studied the genetic diversity using few accessions from Kenya based on random amplified polymorphic DNA (RAPD) and amplified fragment length polymorphism (AFLP), respectively. However, the low sample sizes and the use of dominant markers might have reduced the reliability of the results. Wambulwa et al. (2016) recently characterized the genetic diversity and relationships among 193 tea accessions from Kenya using SSR markers, which revealed that *C. sinensis* var. *assamica* is the most popular tea type in East Africa and harbored the lowest genetic diversity compared to other tea types. Although this study was a significant improvement compared to former studies, it is important to scale up the sampling area to sufficiently cover the African tea growing countries. However, the levels of genetic diversity, and germplasm exchanges of tea cultivars within African countries are still unknown. Given the common practice of germplasm exchanges within Africa, it is expected that *C. sinensis* var. *assamica* would still be the dominant tea type, and to have a low genetic diversity.

Nuclear microsatellite markers (nSSRs) are ubiquitously distributed in the genome, show a co-dominant inheritance, are highly polymorphic, frequently transferable across related species (Chase et al., 1996; Ellegren, 2004; Selkoe and Toone, 2006; Allan and Max, 2010; Fan et al., 2013), and have been widely used to assess the level of genetic variation in many plant species. Variation in chloroplast DNA (cpDNA) have also been used widely in population genetics and for parental identification (Kaundun and Matsumoto, 2011; Paule et al., 2012). CpDNA evolves slowly, has low mutation rates, no recombination, and is usually uniparentally inherited in angiosperms (Wolfe et al., 1987; Clegg and Zurawski, 1992; Mogensen, 1996). It is also known that cpDNA is maternally inherited in Theaceae (Yang et al., 2013). Thus, combined analysis of biparentally inherited nuclear SSR markers and maternally inherited cpDNA could provide an opportunity to investigate the genetic diversity and offer insights into the parental origin of unknown genetic pedigrees of the tea plant created in the process of genetic improvement of tea cultivars in Africa.

In the present study, we use nSSR genotyping, and cpDNA sequencing to investigate the genetic relatedness and breeding patterns of 280 tea accessions collected from eight countries across Africa (including Madagascar). The main objectives were to (1) determine the genetic diversity and structure of African tea at the continental scale, (2) establish the genetic relationships and movement of tea accessions within Africa, and (3) attempt to explore the breeding patterns and uncover differential selection between the TRFCA and TRFK breeding programmes in Africa. The results will provide baseline data that can have significant implications for the improvement of tea cultivars in Africa.

## Materials and methods

### Sample collection and DNA extraction

A total of 280 tea accessions were collected from eight African countries, including Kenya (183 samples), Rwanda (27), Tanzania (10), Malawi (20), South Africa (8), Cameroon (13), Nigeria (4), and Madagascar (15). Out of the total, 183 tea accessions sampled from Kenya represented the germplasm under cultivation in the entire East African region, i.e., Kenya, Uganda, Tanzania, Rwanda, and Burundi. The tea samples from Madagascar were collected from a deserted tea garden (located in Ambositra District) and morphologically identified as *Camellia sinensis* var. *pubilimba* Hung T. Chang, which occurs naturally in South China. The collection target was to cover the historic cultivation areas of southern and eastern Africa as well as the new entrants in western Africa. Details of all accessions are provided in Table S1. Healthy young leaves were sampled from randomly selected plants and immediately stored in silica-gel until DNA extraction. Total DNA was extracted following the cetyltrimethylammonium bromide (CTAB) method as described by Doyle and Doyle (1990).

### nSSR genotyping

Genotyping of the 280 tea accessions was carried out with a total of 23 highly polymorphic nSSRs employed in our previous study (Wambulwa et al., 2016). Visualization and sizing of the SSR fragments was performed using GENEMARKER v.4.0 (SoftGenetics LLC, State College, PA, USA). The genotyping procedure (amplification and fragment size determination) for all samples followed our previous study (Wambulwa et al., 2016). The nSSR data has been deposited at http://dx.doi.org/10.5061/dryad.3g02j in the Dryad Digital Repository.

### cpDNA sequencing

Three fast-evolving plastid intergenic spacer regions (*ndhF-rpl32, trnSGG-trnSr* and *trnSf1-trnGGG*) were sequenced for 84 accessions selected based on results of the nSSR data (Figure S1, Table S1, highlighted in yellow). The 84 accessions represented the various genetic groups in the nSSR NJ tree and origins from the different countries. We also included some known hybrids together with their parents in order to untangle the genetic pedigrees of some specific accessions. Primer sequences for the three regions are provided in Table S2. PCR amplification was carried out on a GeneAmp PCR System 9700 thermal cycler (PerkinElmer, Foster City, CA, USA). The 20 μL PCR mix contained 2 μL of 10 × PCR buffer, 1.6 μL of 25 mM MgCl_2_, 0.4 μL of 10 Mm dNTPs, 0.4 μL of each primer (forward and reverse), 0.15 μL of Taq polymerase (5 units/μL), 1 μL of 40–50 ng/μL of template DNA and 14.05 μL sterile double-distilled water. The PCR conditions included an initial denaturation at 94°C for 3 min, followed by 35 cycles of 30 s at 94°C for template denaturation, 30 s at 50°C for primer annealing, 50 s at 72°C for extension, and followed with an extension step of 10 min at 72°C. PCR products were purified using ExoSAP-IT (GE Healthcare Life Sciences, USA). Purified PCR products were sequenced in both directions with the same primers used for PCR and then analyzed on an ABI 3730xl DNA Sequencer (Applied Biosystems, Forster city, USA). All the cpDNA sequences were deposited in GenBank and the accession numbers are provided in Table S1.

### Data analysis

#### nSSR genotyping

The automated genotyping results were re-checked manually. Genotyping errors such as the presence of null alleles, large allele drop-outs, and stuttering were examined by MICROCHEKER v2.2.3 (Van Oosterhout et al., 2004). The genetic indices, observed heterozygosity (*H*_*O*_), genetic diversity (*H*_S_), and fixation index (*F*) were estimated in Powermarker v3.25 (Liu and Muse, 2005). Genetic distances among countries were calculated in Microsatellite Analyzer (MSA) v4.05 (Dieringer and Schlötterer, 2003), and cluster analyses on the basis of these distances were performed using PHYLIP v3.67 (Felsenstein, 2004) with the neighbor joining (NJ) method. Each branch was tested for reliability by re-sampling with 1000 replications. Finally, the NJ tree was viewed and edited in FigTree v1.4.2 (Rambaut, 2008). Allelic richness was determined by rarefaction analysis in HP-RARE v1.0, a program that compensates for sampling disparity among populations to allow comparison (Kalinowski, 2005). NewHybrids v1.1 beta (Anderson and Thompson, 2002) was used to classify hybrids into their respective classes as detailed in Wambulwa et al. (2016).

We carried out a model-based clustering analysis in STRUCTURE v2.3.4 (Pritchard et al., 2000) to estimate the number of genetic clusters (*K*) without *a priori* knowledge of taxonomy or population location. STRUCTURE was run under the admixture model and correlated allele frequencies with 100,000 generations of “burn-in” and 100,000 Markov chain Monte Carlo (MCMC) iterations for increasing numbers of *K* subdivisions from 1 to 8. Simulations were repeated 20 times for each value of *K*. The optimal number of clusters (*K*) was determined using both L(*K*) (Rosenberg et al., 2001) and Δ*K* (Evanno et al., 2005) methods visualized with STRUCTURE HARVESTER v0.6.92 (Earl and vonHoldt, 2012). A principal co-ordinates analysis (PCoA) was carried out in GenAIEx v6.5 (Peakall and Smouse, 2012).

#### cpDNA sequencing data

DNA sequences were assembled and edited in Sequencher v5.0 (Gene Codes Co., USA). The sequences were aligned with MUSCLE (Edgar, 2004). The edited sequences of the three cpDNA regions were then combined in SequenceMatrix v1.7.8 (Vaidya et al., 2011). Haplotypes of cpDNA sequences were defined using DnaSP v5.10 (Librado and Rozas, 2009), and a haplotype network tree constructed in NETWORK v4.6.1.3 (Fluxus Technology Ltd., Suffolk, UK) with the median-joining method (Bandelt et al., 1999). A Neighbor-joining tree of the 84 accessions was constructed in MEGA v6.0 (Tamura et al., 2013) based on Kimura 2-parameter (K2P) distances and a pairwise deletion model. Geographical mapping of the nSSR genetic groups and haplotype distribution was done sequentially in DIVA-GIS v7.5.0 (http://www.diva-gis.org/) and ArcGIS v10.2.2 (https://www.arcgis.com).

## Results

### Genetic diversity of nSSR markers

No genotyping errors were detected in the nSSR dataset by MICROCHECKER. Only 0.34% missing data were present in the whole dataset due to consistent failures of PCR amplification. After rarefaction analysis, the mean number of alleles per locus across countries varied from 1.59 (*TUGMS2* 157) to 7.83 (*Po*9) (Table S3). Allelic richness ranged from 4.04 (Nigeria) to 6.6 (Cameroon). The overall genetic diversity (*H*_S_) of the African tea accessions was 0.652. At the country level, Kenya exhibited the highest genetic diversity (*H*_S_ = 0.747), followed by Cameroon (0.716), and Rwanda (0.683), with South Africa having the lowest value (0.579). The fixation index (*F*) for the eight countries ranged from −0.17 (South Africa) to 0.112 (Kenya). The summary statistics of the genetic indices are summarized in Table 1.

**Table 1:** **Summary statistics of genetic variation of the 23 SSR loci for 280 tea samples collected from eight African countries**.

**Country**	***N***	***A*_r_**	***H_O_***	***H*_S_**	***F***
Cameroon (CR)	13	6.6	0.686	0.716	0.058
Kenya (KN)	183	6.49	0.663	0.747	0.112
Madagascar (MD)	15	4.42	0.556	0.614	0.097
Malawi (MW)	20	5.06	0.726	0.642	−0.063
Nigeria (NG)	4	4.04	0.652	0.621	−0.103
Rwanda (RD)	27	5.65	0.739	0.683	−0.063
South Africa (SA)	8	4.74	0.630	0.579	−0.170
Tanzania (TZ)	10	4.87	0.700	0.609	−0.152
Total/Mean	280		0.6691	0.652	−0.036

*N, Sample size; A_r_, Average allelic richness over loci; H_O_, Observed heterozygosity; H_S_, Genetic diversity; F, Fixation index*.

### Genetic structure and clustering patterns of nSSR data

**Figure 1:**
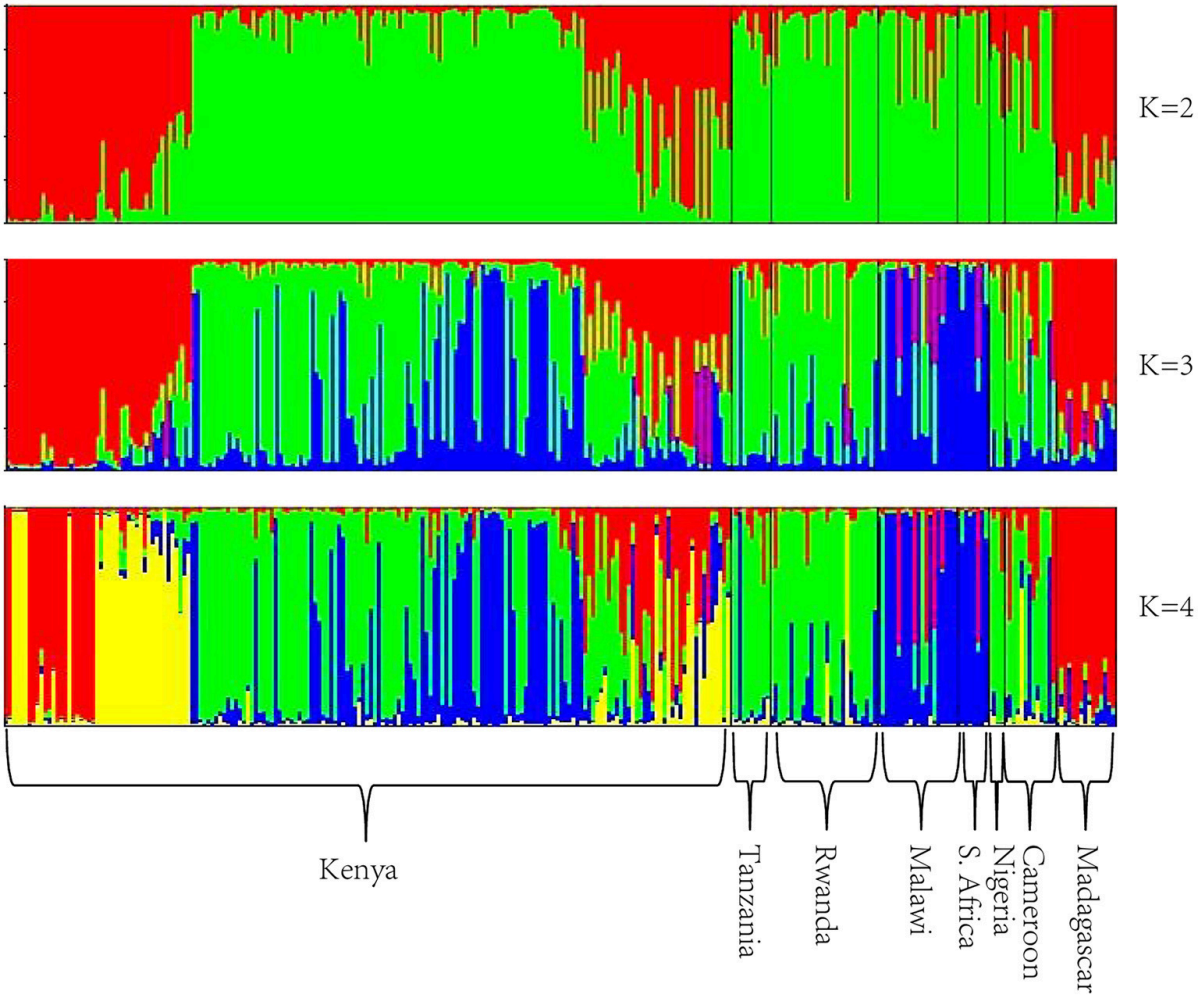
**Bayesian assignment of probabilities using STRUCTURE based on 23 nSSR loci of the 280 tea accessions**.

**Figure 2:**
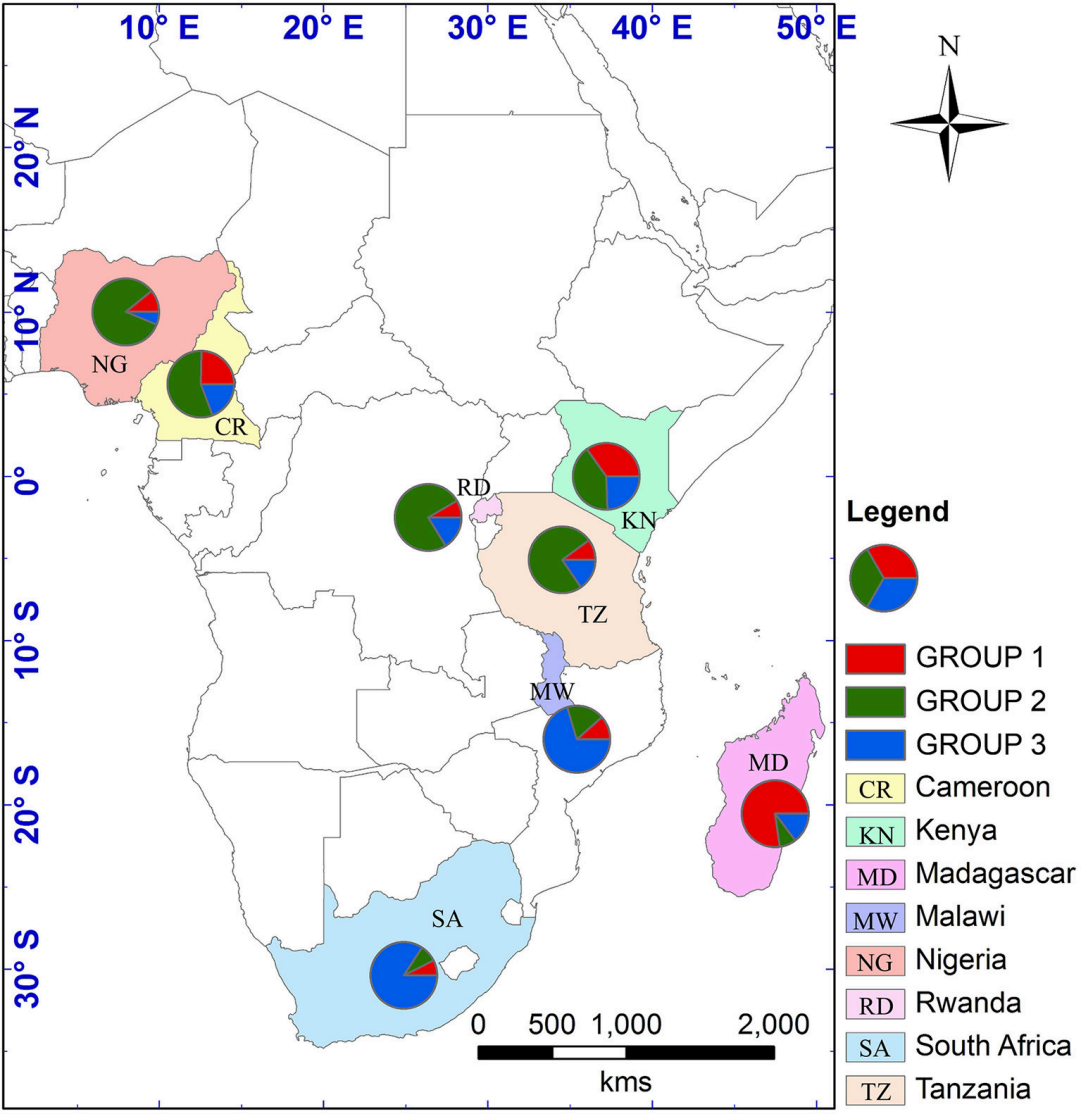
**Geographical distribution of nSSR genetic groups considered following STRUCTURE analysis at ***K*** = 3**. Each country is depicted as a pie chart with the proportional membership of its alleles to each one of the three groups. A shape file with genotype proportions in the different countries was generated in DIVA-GIS v7.5.0.0 (http://www.diva-gis.org/). The shape file was then used to generate the map in ArcGIS v10.2.2 (http://www.esri.com/).

The optimum value for *K* was 2, suggested by both L(*K*) and Δ*K* approaches (Figure S2). The genetic clustering of the 280 tea accessions is shown for *K* = 2, 3, and 4 (Figure 1). At *K* = 2, two genetic groups were defined, which corresponded to the *C. sinensis* var. *sinensis* and *C. sinensis* var. *assamica* groups found in our previous study (Wambulwa et al., 2016). *Camellia sinensis* var. *sinensis* (in red) included samples from Madagascar and some accessions from Kenya, while *C. sinensis* var. *assamica* (in green) contained most accessions from Kenya and those from the remaining six countries. Within both groups, many accessions possessed a mix of genetic material from both groups (representing “mosaic” genotypes). At *K* = 3, a new group (in blue) was separated from the *C. sinensis* var. *assamica* group, and included accessions mainly from Malawi, South Africa, and Kenya, while overall more accessions showed a “mosaic” genotype. At *K* = 4, some accessions from Kenya formed an additional group (in yellow) separated from the red group (*C. sinensis* var. *sinensis*; Figure 1). Geographical mapping of the genetic groups was based on the posterior probabilities at *K* = 3, because at this *K*-value, *C. sinensis* var. *assamica* (which constituted the majority of African tea) separated into two groups which are mainly distributed in southern and East Africa respectively (Figure 2).

The NJ tree showed three main clusters (Figure S1). The basal cluster (Cluster I) was comprised of seven accessions from Kenya (TRFK 91/1, TRFK 91/2, TRFK 306/1, TRFK 306/2, TRFK 306/3, TRFK 306/4, and TRFK 830/5). Cluster II included three sub-clusters that contained a total of eleven accessions from Malawi, Kenya, Nigeria, and South Africa. The pioneer accession for the introduction of the tea plant in Malawi (SFS 150) and its counterparts in Kenya and South Africa formed one of the sub-clusters. The third cluster (Cluster III) consisted of the remaining 262 accessions which were grouped into 34 polytomic sub-clusters.

The clustering behavior of tea accessions in the PCoA plot highlighted the distinction between the southern and East African gene pools, a pattern that was generally consistent with the NJ tree results (Figures S1, S3). Most accessions from Malawi and South Africa grouped into a single cluster including three accessions from Rwanda (35/49, 22/17, and K/32) and one from Tanzania (TRIT 201/82). The Kenyan accessions, TRFK 31/11 and TRFK 18/16, formed a single cluster together with 10 accessions from Rwanda. Five Tanzanian tea accessions grouped together with three Kenyan accessions (TRFK 430/90, TRFK 420/13, and TRFK 14/1). Some Rwandan accessions, e.g., RW/G539, RW/SR/2B1, and RW/SR/2B1/49, clustered separately with Kenyan accessions.

In fact, no clear country-specific genetic cluster was observed, except perhaps for Madagascar, albeit this including one accession from Cameroon (CL H81/22). In addition, in six cases accessions from different countries but with similar codes fell in the same subclusters: PC 108 (Malawi) and PC 108, 15/4PC 108 (South Africa); PC 81 (Malawi), 16/4PC 81 (South Africa), and AHP PC 81 (Kenya); SFS 150 (Malawi), 16/4SFS 150 (South Africa), and TRFCA SFS 150 (Kenya); BBK 35 (Kenya) and Var BB 35 (Nigeria); TRFK 6/8 (Kenya) and Var 68 (Nigeria); and SFS 204 (Malawi) and 16/4SFS 204 (South Africa).

**Figure 3:**
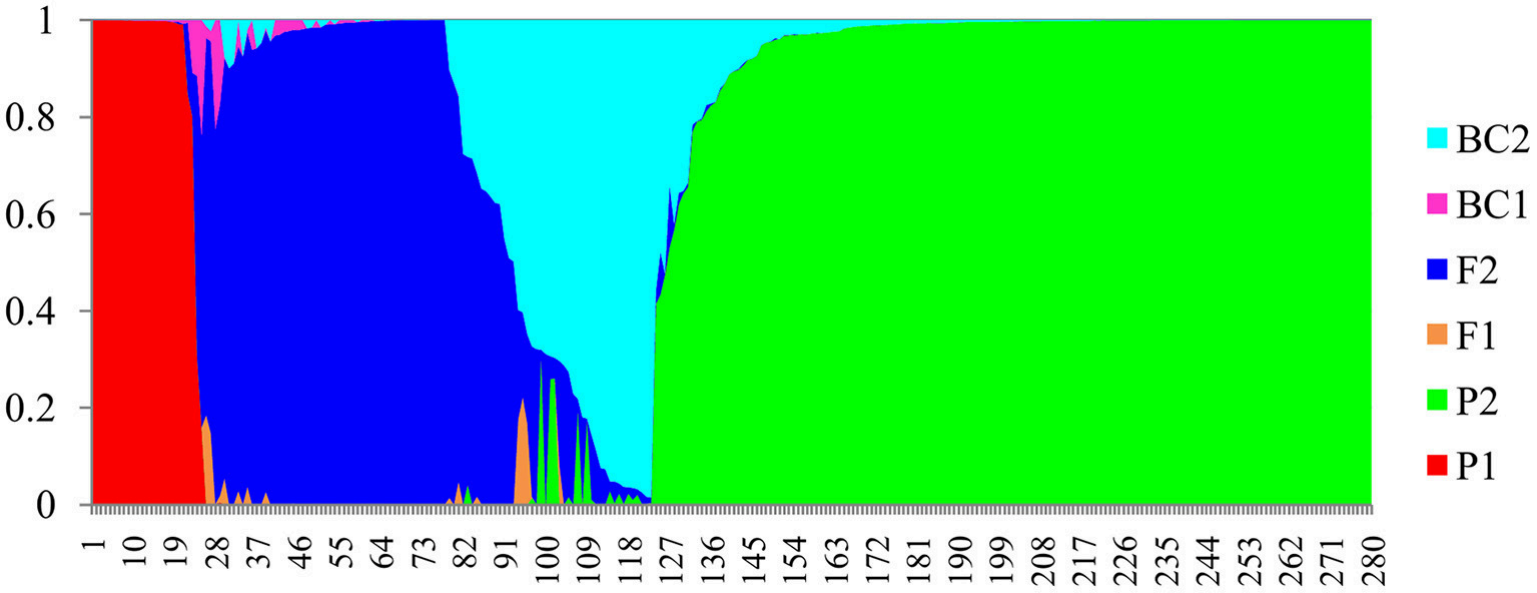
**Bayesian assignment of probabilities in NewHybrids for 280 tea accessions from Africa**. The defined categories are parent 1 (P1), parent 2 (P2), first filial generation (F1), second filial generation (F2), backcross to P1 (BC1), and backcross to P2 (BC2).

In the NewHybrids analysis, a total of 22 accessions from Kenya were assigned P1 (parent 1), and 146 accessions assigned P2 (parent 2). The two parent assignations corresponded to *C*. *sinensis* var. *sinensis* and *C. sinensis* var. *assamica*, respectively, (Figure 3). For the remaining 112 accessions, a hybrid origin was suggested, including 70 F2s (second filial generation), one BC1 (backcross to parent 1), and 41 BC2s (backcross to parent 2), but no F1 (first filial generation) generation hybrid was detected. All 15 samples from Madagascar were assigned F2 hybrids status. Details of the distribution of the various parental and hybrid classes are provided in Tables S1, S4.

#### cpDNA sequencing and relationships of haplotypes

**Figure 4:**
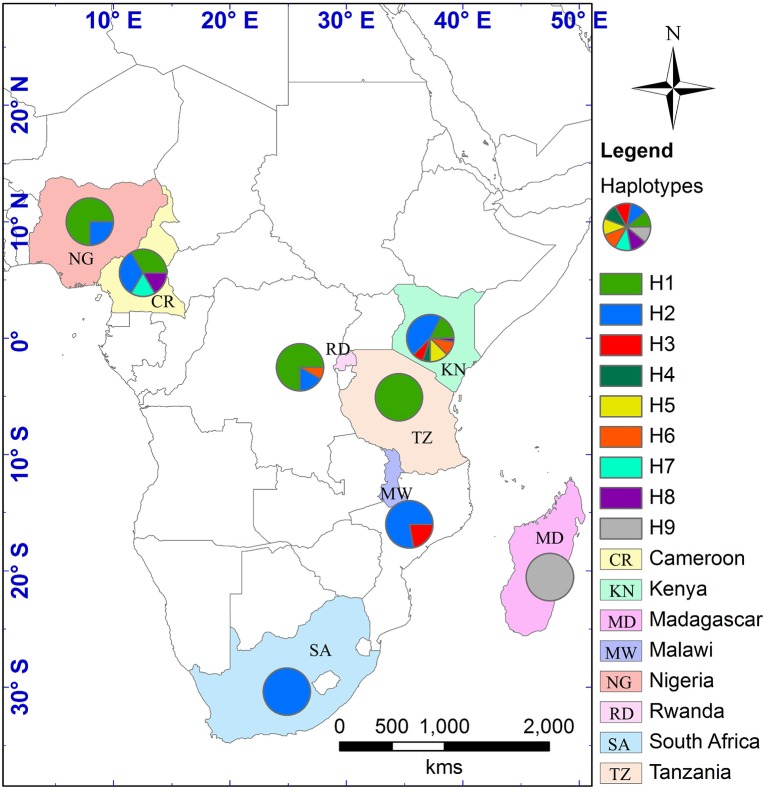
**Geographical distribution of the nine cpDNA found in the 280 samples across eight countries in Africa**. The map was generated using DIVA-GIS and ArcGIS software as described in Figure 2.

**Figure 5:**
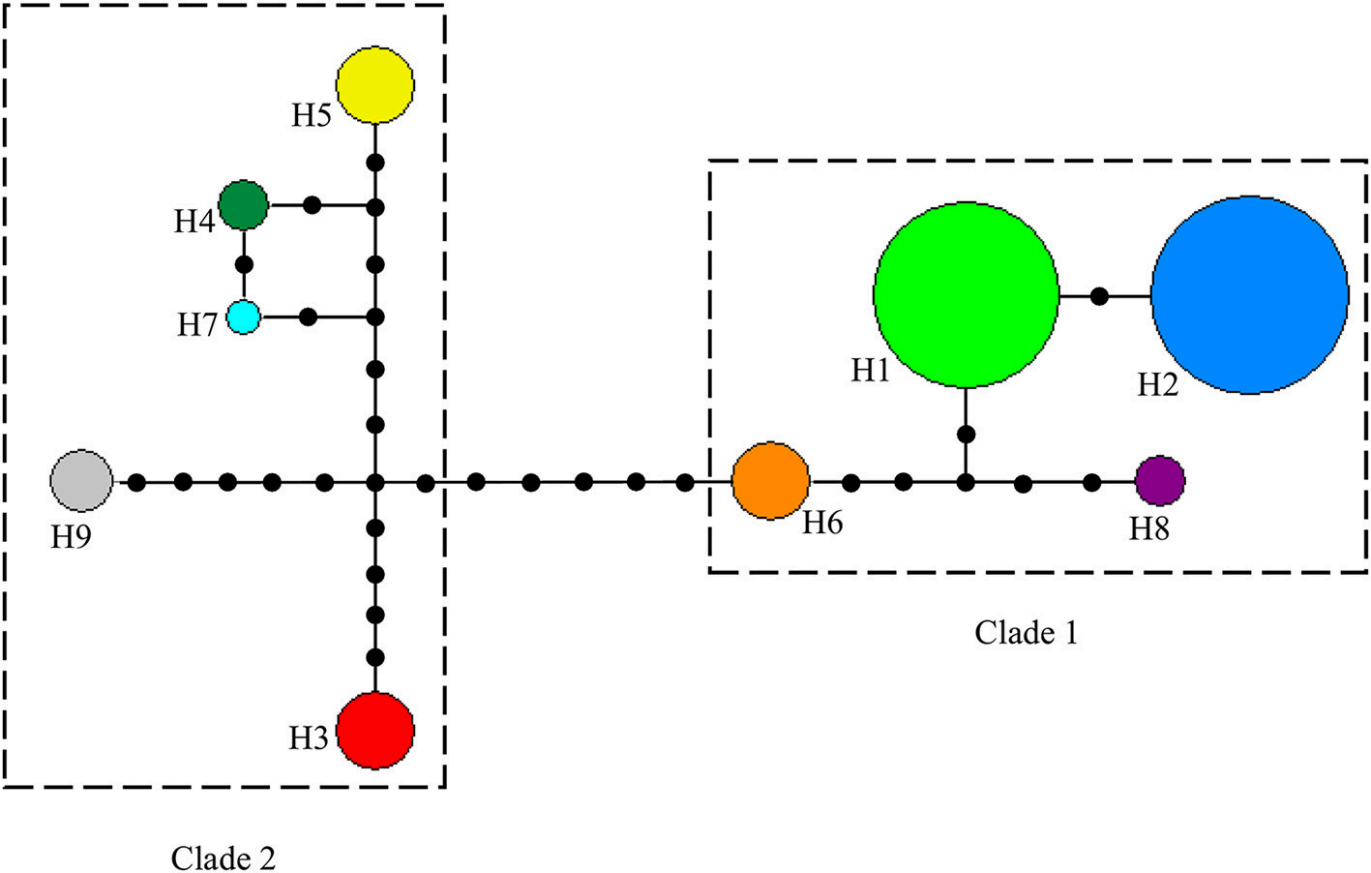
**Median-joining haplotype network based on three combined cpDNA regions of 84 tea samples from eight African countries**. Broken lines delineate closely related haplotypes. Circles in colors denote different haplotypes (H1-H9), and the size of each circle is proportional to the number of accessions sharing that particular haplotype. Each branch between haplotypes denotes a mutational step. The small black circles represent independent mutation events converging on a shared haplotype.

**Figure 6:**
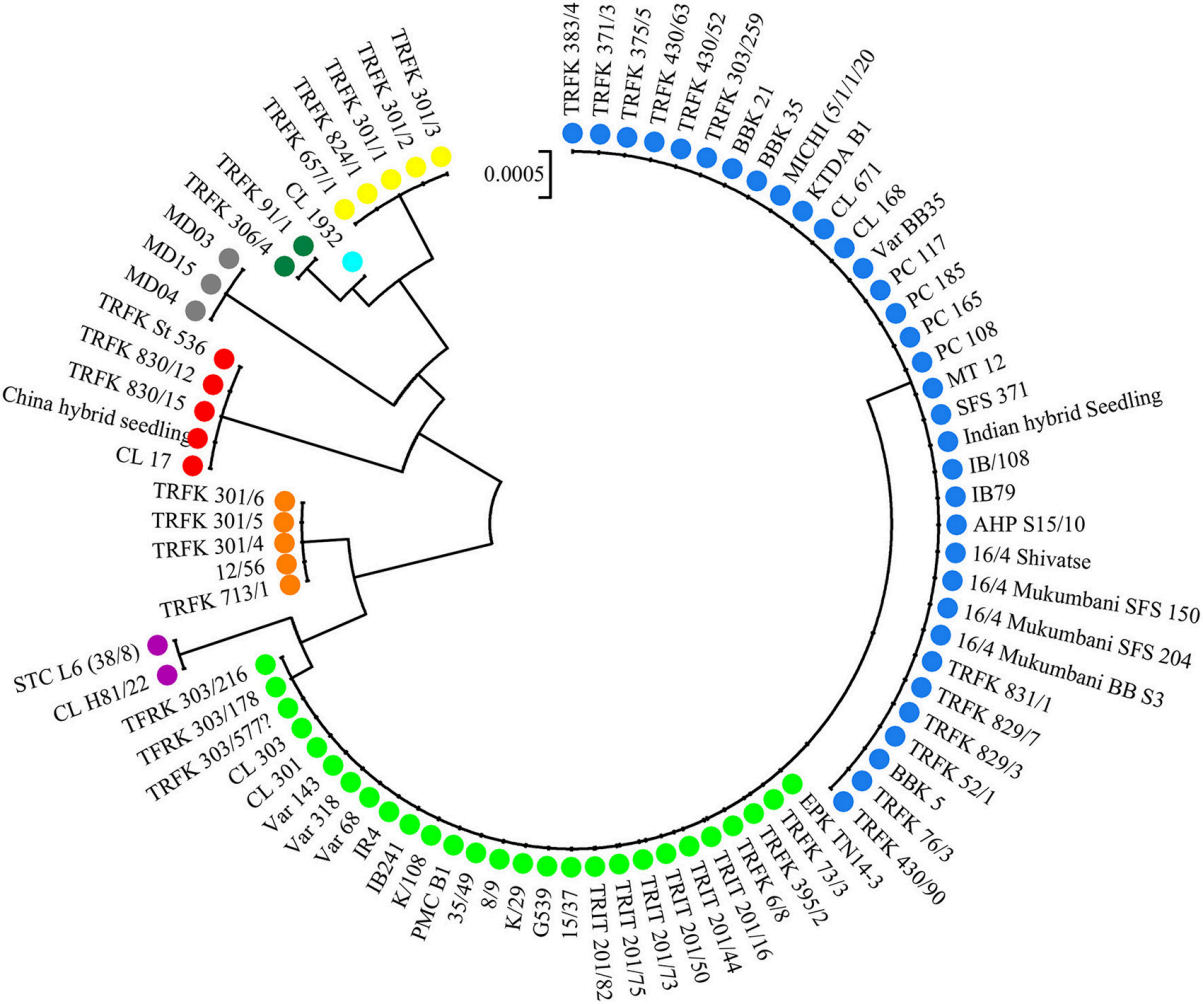
**Neighbor joining tree of 84 tea samples**. Each color represents a particular haplotype and corresponds to colors in Figure 5.

The alignment of the three cpDNA regions of *ndhF-rpl32, trnSGG-trnSr*, and *trnSf1-trnGGG* was 661 bp, 647 bp, and 601 bp in length, respectively. The concatenated matrix of the three cpDNA regions was 1909 bp long and contained 42 polymorphic sites (2.2%). A total of nine haplotypes (H1–H9) were identified for the 84 African tea accessions. Four out of these were private to particular countries, two found in Kenya, and one each in Cameroon and Madagascar, respectively. The Kenyan samples had the highest number of haplotypes (7), followed by Cameroon (4) then Rwanda (3) (Figure 4, Table S5). The haplotype network and NJ tree revealed similar relationships (Figures 5, 6). The nine haplotypes could be divided into two clades. Haplotypes H1, H2, H6, and H8 formed Clade 1, which was associated with *C. sinensis* var. *assamica*. In this clade, H1 and H2 were the two most dominant haplotype with one mutation step between them, with H1 mainly occurring in East and West Africa, while H2 was dominant in southern Africa. Clade 2 consisted of the remaining haplotypes and was associated with *C*. *sinensis* var. *sinensis*; H3 was specific to *C*. *sinensis* var. *sinensis* while H9 was present in all samples from Madagascar, corresponding to *C*. *sinensis* var. *pubilimba*. Haplotypes H4, H5, and H7 may represent a distinct lineage of tea; for instance H4 is shared with accession TRFK 91/1 which is *Camellia irrawadiensis*, a wild relative of the tea plant. Overall, H1 and H2 showed the highest proportions among all accessions with 32.14 and 40.48%, respectively. Comparison with the NewHybrids results showed that all accessions in Clade 1 (except TRFK 306/4) were of P2, F2, and BC2 origin, while all accessions with H3 and H9 were assigned P1 and F2 (H9) status.

The accessions CL 671 and China Hybrid Seedling fell in one subcluster based on the nSSR data (Figure S1), but had different cpDNA haplotypes of H2, and H3, respectively, (Table S5). TRFK 430/90 and TRIT 201/75, and CL H81/22 and the Madagascan accessions, showed the same pattern. On the other hand, the three known hybrids between TRFCA SFS 150 and EPK TN14-3 (TRFK 430/52, TRFK 430/63, and TRFK 430/90) from Kenya shared the same haplotype with SFS 150.

## Discussion

### Genetic diversity and clustering

Genotyping and assessment of genetic diversity is an important prerequisite for the identification of core germplasm collections for conservation and optimizing crop improvement efforts (Fu, 2015). This is the first study to assess the genetic diversity of the tea plant across all the main tea-growing countries in Africa. We found that the genetic diversity of African tea (0.652) is much lower than that of Asia (0.86) calculated over 788 accessions from 14 countries, with both studies using 23 nSSR markers (Taniguchi et al., 2014). The higher diversity estimated for this region possibly results from the wider genetic sampling from the cradle of the tea plant in China and India (Hashimoto and Takashi, 1978; Hashimoto, 2001), which inherently possess higher levels of genetic diversity (Yao et al., 2012). Interestingly, our recent study found higher genetic variation among the East African tea accessions (0.717), probably because of the *ex situ* germplasm bank of tea plants in Kenya which contains accessions that are genetically highly disparate and assembled from various geographical origins (Wambulwa et al., 2016).

In a separate study, however, the Kenyan tea samples harbored the lowest genetic diversity when compared to Chinese and Japanese teas (Yao et al., 2008). The low variation could be attributed to the relatively short history of Kenyan tea plantations, the small sample size (only four accessions included) and the dominant markers used (ISSR). Sample size bias may be a potential confounding factor of genetic diversity estimates. In this study, we determined allelic richness based on rarefaction analysis, thus eliminating the problem of sample size disparity. Cameroon also showed the highest allelic richness (*A*_r_ = 6.6) despite its relatively small sample size, which possibly resulted from the diverse genetic origin of the tea cultivars in this country, as evidenced by the existence of five haplotypes including a private one. Unsurprisingly, among the African accessions, Kenya also exhibited a high genetic variation perhaps due to the diversity of geographic origins of the samples (Wambulwa et al., 2016).

Our results highlight the importance of the combination of differently inherited markers to clarify the breeding patterns of the tea plants in Africa. For example, TRFK 430/52, TRFK 430/63, and TRFK 430/90 are known to be hybrids between TRFCA SFS150 and EPK TN14-3 but without information on the direction of the cross. The haplotype analysis identified TRFCA SFS 150 to be the maternal parent, because the three hybrids shared its haplotype. In addition, Madagascan tea accessions could not be separated from *C*. *sinensis* var. *sinensis* accessions from Kenya by nSSR data. However, the cpDNA data could separate them into distinct haplotypes H3 and H9. This might indicate that the tea cultivars from Madagascar are of likely hybrid origin between *C*. *sinensis* var. *sinensis* (paternal) and *C*. *sinensis* var. *pubilimba* (maternal) native to South China, and probably introduced independently of those from mainland Africa. Accessions CL671 and China Hybrid Seedling also have different haplotypes but a similar nuclear genetic composition, implying that these accessions are also hybrids of BC or F2 (or later) generation with diverse maternal parents but probably sharing the same paternal parent, which was confirmed in our NewHybrids analysis.

### Genetic relationships and germplasm exchange of tea accessions within Africa

Our results showed that African tea germplasm is genetically admixed and shared among countries, indicating that the tea accessions were often exchanged among different countries. For instance, the clustering patterns in the NJ tree suggested that the Rwandan accessions RW/35/49, RW/22/17, and RW/K/32 and the Tanzanian accession TRIT 201/82 might have been introduced from South Africa, while the Kenyan accessions TRFK 430/90, TRFK 420/13, and TRFK 14/1 possibly originated from Tanzania. The typically southern accessions of the SFS and PC series (with haplotype H2) are dominant in southern Africa and with low frequency in Kenya, Rwanda, Cameroon, and Nigeria, suggesting germplasm movement from the southern Africa region (Malawi and South Africa) northwards. Moreover, historical records showed that the southern breeding program (TRFCA) is the oldest in Africa (Anonymous, 1962). We therefore deduced that some of the pioneer germplasm or offspring thereof might have been moved northwards from southern Africa to East Africa and eventually to West Africa. Unique haplotypes were found in Cameroon, Kenya, and Madagascar, which suggested possibly independent germplasm exchanges and introductions from countries outside of Africa. Germplasm exchanges among countries may be an important strategy for tea or crop improvement in general. International exchange of crop germplasm is essential both for ensuring food security and for promoting research relations among the countries involved (Singh et al., 2012). In the face of the threats from climate change, interdependency among countries for genetic material of crop plants is expected to accelerate the development of new varieties for better adaptation to future climate challenges (FAO, 2015b). However, germplasm exchange should be exercised cautiously and aided by genetic fingerprinting methods, or otherwise problems of nominal redundancies in the accession databases may arise, exacerbated by the tendency of some countries to adopt different names for the same accession (Kisha and Cramer, 2011). In the present study, we did indeed find that accessions with similar names from different countries clustered together (e.g., SFS 150 from Malawi, 16/4SFS 150 from South Africa, and TRFCA SFS 150 from Kenya), suggesting that they are the same accession with slightly modified identifiers.

The NewHybrids analysis here showed similar results to our previous study on East African tea accessions (Wambulwa et al., 2016). Cultivars of *C*. *sinensis* var. *assamica* are the most popular and dominant tea plants in Africa. The popularity of *C*. *sinensis* var. *assamica* might be linked to its faster growth, ease of harvest and higher yields (Seurei, 1996). Accessions of *C*. *sinensis* var. *sinensis* on the other hand, are mainly held in germplasm banks as a breeding resource for hybridization, due to their lower yield because of the small leaves that are difficult to pluck. The introduction of machine harvesting, however, may result in commercialization of some of these cultivars for green and specialty tea processing in the future. Hybrid cultivars made up a high proportion of 62.5 (F2) and 36.6% (BC2) of the accessions. In these accessions, *C*. *sinensis* var. *assamica* contributed much more than *C*. *sinensis* var. *sinensis* as maternal parent. The distinct genetic group formed by the hybrids (Figure 1, in yellow) indicated that these cultivars are possibly fixed for some alleles linked to adaptation to local environments during their breeding history.

### Breeding centers and conservation of tea germplasm

Southern Africa (TRFCA) and East Africa (TRFK) are recognized as the oldest breeding centers in Africa (Matheson and Bovill, 1950; Anonymous, 1962). Our results of both nSSR and cpDNA haplotype analysis basically reflect the activities of the two breeding centers on mainland Africa. Two distinct genetic groups were defined for *C*. *sinensis* var. *assamica* accessions, a southern group (in blue) and an eastern group (in green; Figures 2, 4). Although the haplotypes H1 and H2 differed only by a single mutation, H1 was associated with the pioneer selection TRFK 6/8 from East Africa, and H2 is preponderant in pioneer selections SFS 371, SFS 150, and SFS 204 from southern Africa (South Africa and Malawi; Figure 4). We concluded that TRFCA (southern Africa) and TRFK (East Africa) are the two main tea breeding centers for *C*. *sinensis* var. *assamica* in Africa, and have independently selected cultivars for specific traits. The occurrence of two distinct gene pools of *C*. *sinensis* var. *assamica* could be attributed to breeders preferences and the subsequent accumulation of divergently selected traits from distinct genetic resources to breed new preeminent cultivars.

Yield and quality are the universal traits of focus in tea breeding (Mondal, 2014). However, climate adaptation-associated traits might have played an important role in differentiating the accessions of the two breeding centers. Such traits include drought tolerance, reduced winter dormancy, hail/frost resistance, water logging tolerance, and cold hardiness. Southern Africa has a transition zone of sub-tropical and temperate climates, with desert or semi-arid regions centered in Namibia and Botswana, while eastern (equatorial) Africa has a tropical climate with a relatively high altitude and high annual precipitation. Recent studies on the tea plant have demonstrated that variations in climatic conditions can significantly affect profiles of secondary metabolite such as catechins and methylxanthines (Ahmed et al., 2014; Kowalsick et al., 2014; Larson, 2015) which directly influence tea quality. Going forward, it will be important for tea breeders to make use of niche modeling and climate data as a novel strategy for developing locally well adapted tea cultivars from divergent gene pools.

*Camellia sinensis* var. *assamica* is the main tea type under cultivation in Africa, a situation that may compromise the ability of the germplasm to adapt to biotic and abiotic challenges as a result of its relative genetic homogeneity. It is therefore important to maintain diverse genetic germplasm lineages. In the present study, several divergent haplotypes (H4, H5, H7, and H9) not derived from the tea plant were observed in the African germplasm, and may represent distinct gene pools, such as *C*. *sinensis* var. *assamica* in China that was recently defined as a new genetic entity (Meegahakumbura et al., 2016), and wild relatives of the tea plant. In fact, H4 represented a haplotype derived from *C*. *irrawadiensis*, native to Myanmar, which might have been introduced to breed for the presence of anthocyanin (purple) pigments in certain tea accessions (Kamunya et al., 2009). These accessions may be highly valuable resources for tea breeding in the future. Thus, it is very important to conserve such elite genetic stock as resources diversifying the African tea gene pool to be flexible to adjust to future directions in tea breeding. The phenomenon of differential selection by breeders is not limited to our study. A similar observation was made in maize (Van Heerwaarden et al., 2012), sorghum (Morris et al., 2013), wheat (Cavanagh et al., 2013), and rice (Xie et al., 2015) where accessions originating from different breeding programmes showed high differentiation. It has been shown that locally selected material collectively possesses a high diversity and potential for local adaptation (Ravigné et al., 2009), which will be very important for mitigating the adverse influence of climate change and other environmental abiotic and biotic challenges.

## Conclusions

We investigated the genetic diversity and relationships for 280 African tea accessions based on 23 polymorphic nSSR markers and three cpDNA sequence regions. Our results indicated that African tea has a relatively low genetic diversity, and *C*. *sinensis* var. *assamica* is the main tea type under cultivation and contributes more in tea breeding improvement programs in Africa. Internal germplasm exchange and movement among countries within Africa is evident, with Southern Africa (TRFCA) and East Africa (TRFK) possibly being the two major tea breeding centers in Africa. This study represents the first step toward effective utilization of differently inherited molecular markers for exploring the breeding improvement of African tea. The findings will be important for planning the exploration, utilization, and conservation of tea germplasm for future breeding efforts in Africa.

## Data accessibility

The nSSR data set supporting the results in this study are available from the Dryad Digital Repository: http://dx.doi.org/10.5061/dryad.3g02j. The cpDNA sequence data in this study are deposited in GenBank under accession numbers KX584101-KX584352 as shown in Table S1.

## Author contributions

MW, SK, and AM collected plant material. MW and MKM performed the experiments. MW, MM, SR, JL, and LG analyzed and interpreted the data. MW, SK, MM, DL, and LG wrote and revised the manuscript. DL, LG, JX, and AM conceived and designed the study. All authors reviewed and approved the final manuscript.

### Conflict of interest statement

The authors declare that the research was conducted in the absence of any commercial or financial relationships that could be construed as a potential conflict of interest. The reviewer LB and handling Editor declared their shared affiliation, and the handling Editor states that the process nevertheless met the standards of a fair and objective review.

## Abbreviations

AFLP: Amplified fragment length polymorphism
BC1: Backcross to parent 1
BC2: Backcross to parent 2
cpDNA: Chloroplast DNA
CTAB: Cetyltrimethylammonium bromide
F1: First filial generation
F2: Second filial generation
FS: Field selections
H: Haplotype
MCMC: Markov chain Monte Carlo
nSSR: Nuclear simple sequence repeats
P1: Parent 1
P2: Parent 2
PCoA: Principal co-ordinates analysis
RAPD: Random amplified polymorphic DNA
SFS: Swazi field selection
TRFCA: Tea Research Foundation of Central Africa
TRFK: Tea Research Foundation of Kenya.
