# Supplementary material for: Insights into the Genetic Relationships and Breeding Patterns of the African Tea Germplasm Based on nSSR Markers and cpDNA Sequences
Source: Front Plant Sci. 2016 Aug 30;7:1244. doi: 10.3389/fpls.2016.01244 (PMC5004484; doi:10.3389/fpls.2016.01244)
Supplement: Supplementary file 2 [file Table2.docx]

**Table S2:** Primer sequences of the three regions of cpDNA

| **Primer name** | **Sequence 5'-3'** |
| --- | --- |
| ndhF | GAA AGG TAT KAT CCA YGM ATA TT |
| rpl32-R | CCA ATA TCC CTT YYT TTT CCA A |
| trnSGG | AGA TAG GGA TTC GAA CCC TCG GT |
| trnSr | TTA CCA CTA AAC TAT ACC CGC |
| trnSf1 | CCT CTC AAT GAC AGA TTC G |
| trnGGG | GTA GCG GGA ATC GAA CCC GCA TC |
