# Supplementary material for: Insights into the Genetic Relationships and Breeding Patterns of the African Tea Germplasm Based on nSSR Markers and cpDNA Sequences
Source: Front Plant Sci. 2016 Aug 30;7:1244. doi: 10.3389/fpls.2016.01244 (PMC5004484; doi:10.3389/fpls.2016.01244)
Supplement: Supplementary file 4 [file Table4.docx]

**Table S4**: Assignment of accessions to various hybrid classes based on posterior probabilities in the programme NewHybrids.P1 First parent, P2 Second parent, F1 First filial generation, F2 Second filial generation, BC1 Backcross to parent 1, BC2 Backcross to parent 2.

| Country | P1 | P2 | F1 | F2 | BC1 | BC2 |
| --- | --- | --- | --- | --- | --- | --- |
| Cameroon | 0 | 6 | 0 | 3 | 0 | 4 |
| Kenya | 22 | 89 | 0 | 47 | 1 | 24 |
| Madagascar | 0 | 0 | 0 | 15 | 0 | 0 |
| Malawi | 0 | 14 | 0 | 3 | 0 | 3 |
| Nigeria | 0 | 3 | 0 | 1 | 0 | 0 |
| Rwanda | 0 | 22 | 0 | 1 | 0 | 4 |
| South Africa | 0 | 6 | 0 | 0 | 0 | 2 |
| Tanzania | 0 | 6 | 0 | 0 | 0 | 4 |
| **Total** | **22** | **146** | **0** | **70** | **1** | **41** |
