# Supplementary material for: Insights into the Genetic Relationships and Breeding Patterns of the African Tea Germplasm Based on nSSR Markers and cpDNA Sequences
Source: Front Plant Sci. 2016 Aug 30;7:1244. doi: 10.3389/fpls.2016.01244 (PMC5004484; doi:10.3389/fpls.2016.01244)
Supplement: Supplementary file 5 [file Table5.docx]

**Table S5:** Details of the 9 tea haplotypes based on sequences of three cpDNA regions for 84 African tea accessions.

| **Haplotype** | **Accessions** | **Country** |
| --- | --- | --- |
| H1 | EPK TN14-3, TRFK 73/3, TRFK 395/2, TRFK 6/8, TRIT 201/16, TRIT 201/44, TRIT 201/50, TRIT 201/73, TRIT 201/75, TRIT 201/82, 15/37, G539, K/29, 8/9, 35/49, PMCB1, K/108, IB241, IR4, Var 68, Var 318, Var 143, CL 301, CL 303, TRFK 303/577, TRFK 303/178, TRFK 303/216 | Cameroon, Kenya, Nigeria, Rwanda, Tanzania |
| H2 | TRFK 430/90, TRFK 76/3, BBK 5, TRFK 52/1, TRFK 829/3, TRFK 829/7, TRFK 831/1, 16/4 Mukumbani BB S3, 16/4 Mukumbani SFS 204, 16/4 Mukumbani SFS 150, 16/4 Shivatse, AHP S15/10, IB79, IB/108, Indian hybrid Seedling, SFS 371, MT 12, PC 108, PC 165, PC 185, PC 117, Var BB35, CL 168, CL 671, KTDA B1, KTDA MICHI (5/1/1/20), BBK 35, BBK 21, TRFK 303/259, TRFK 430/52, TRFK 430/63, TRFK 375/5, TRFK 383/4, TRFK 371/3 | Kenya, South Africa, Rwanda, Malawi Nigeria, Cameroon |
| H3 | TRFK St 536, TRFK 830/12, TRFK 830/15, CL 17, China hybrid seedling | Kenya, Malawi |
| H4* | TRFK 306/4, TRFK 91/1 | Kenya |
| H5* | TRFK 657/1, TRFK 824/1, TRFK 301/1, TRFK 301/2, TRFK 301/3 | Kenya |
| H6 | TRFK 713/1, 12/56, TRFK 301/4, TRFK 301/5, TRFK 301/6 | Kenya, Rwanda |
| H7* | CL 1932 | Cameroon |
| H8 | CL H81/22, STC L6(38/8) | Cameroon, Kenya |
| H9* | MD03, MD04, MD15 | Madagascar |

* denotes private haplotypes
