# Supplementary material for: Insights into the Genetic Relationships and Breeding Patterns of the African Tea Germplasm Based on nSSR Markers and cpDNA Sequences
Source: Front Plant Sci. 2016 Aug 30;7:1244. doi: 10.3389/fpls.2016.01244 (PMC5004484; doi:10.3389/fpls.2016.01244)
Supplement: Supplementary file 6 [file Image1.pdf]

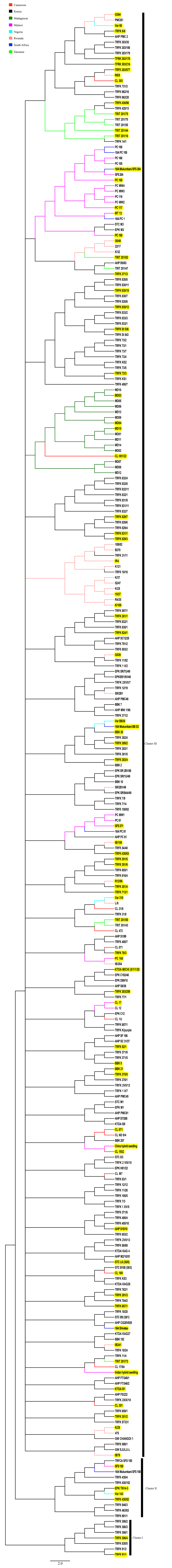

Figure S1: An unrooted neighbour joining tree based on Nei's genetic distances for 280 tea accessions from 8 African countries
