# Supplementary material for: Insights into the Genetic Relationships and Breeding Patterns of the African Tea Germplasm Based on nSSR Markers and cpDNA Sequences
Source: Front Plant Sci. 2016 Aug 30;7:1244. doi: 10.3389/fpls.2016.01244 (PMC5004484; doi:10.3389/fpls.2016.01244)
Supplement: Supplementary file 7 [file Image2.pdf]

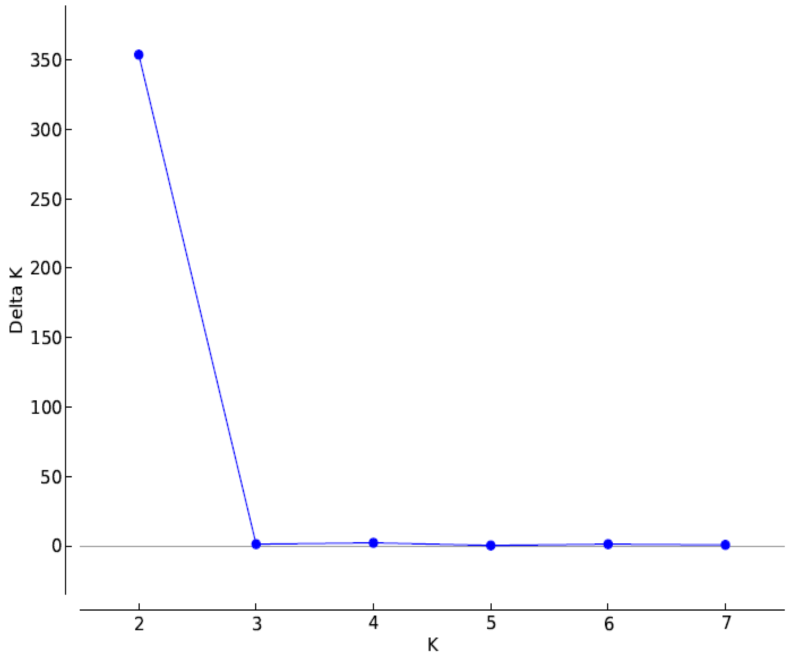

A

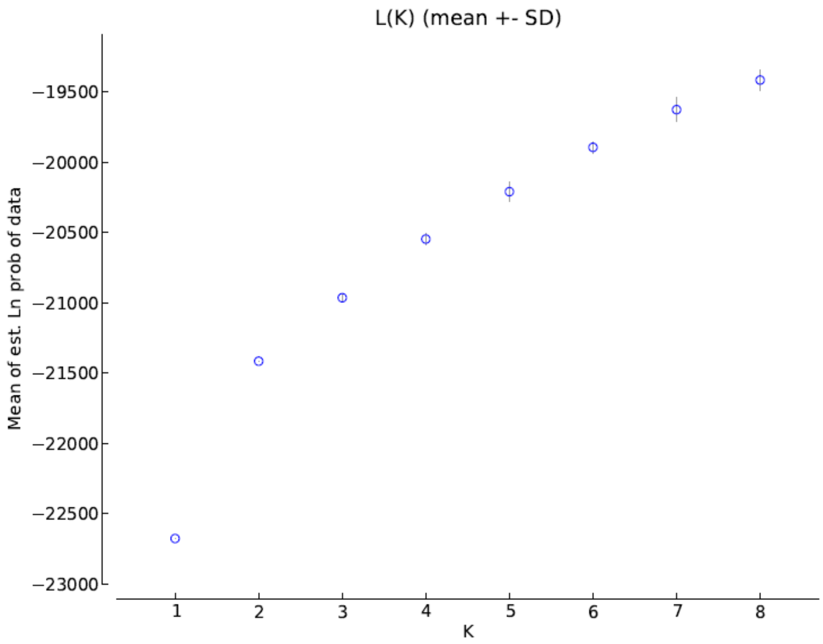

B

Figure S2: Estimated *ΔK* (A) and L(*K*) (B) of 280 tea accessions from Africa over eight runs for each *K* value
