# Supplementary figures and images for: Insights into the Genetic Relationships and Breeding Patterns of the African Tea Germplasm Based on nSSR Markers and cpDNA Sequences
Source: Front Plant Sci. 2016 Aug 30;7:1244. doi: 10.3389/fpls.2016.01244 (PMC5004484; doi:10.3389/fpls.2016.01244)

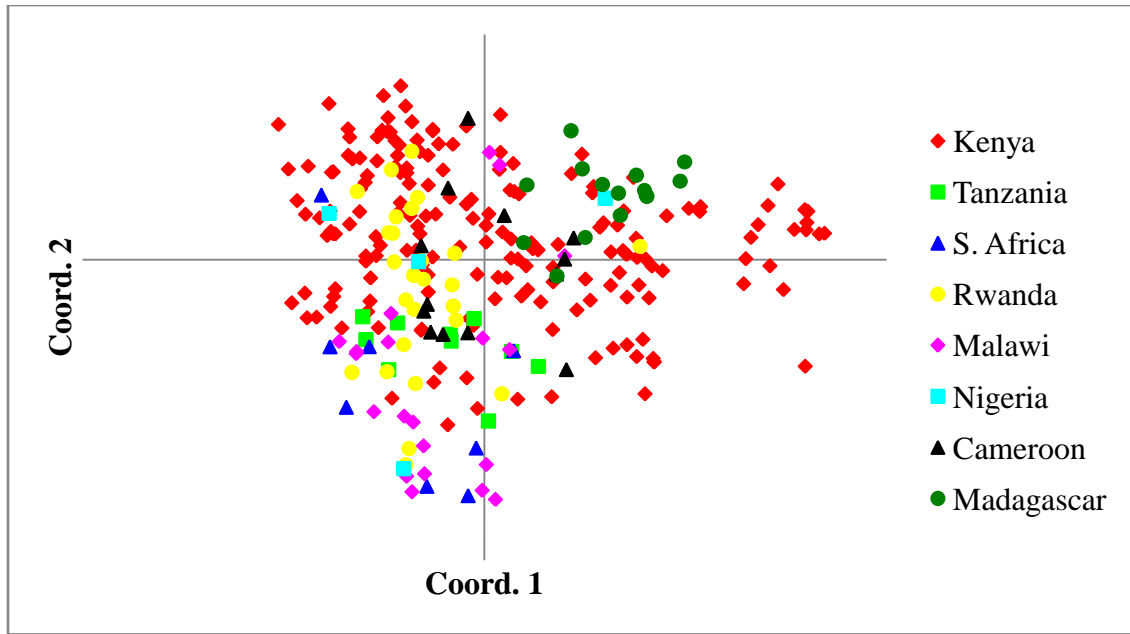

**Figure S3:** Principal coordinates analysis (PCoA) of 280 tea accessions genotyped with 23 nSSR markers.

Supplement: Supplementary file 8 [file Image3.pdf]
